# Blood Cadmium Levels and Incident Cardiovascular Events during Follow-up in a Population-Based Cohort of Swedish Adults: The Malmö Diet and Cancer Study

**DOI:** 10.1289/ehp.1509735

**Published:** 2015-10-30

**Authors:** Lars Barregard, Gerd Sallsten, Björn Fagerberg, Yan Borné, Margaretha Persson, Bo Hedblad, Gunnar Engström

**Affiliations:** 1 Occupational and Environmental Medicine, Sahlgrenska University Hospital and University of Gothenburg, Gothenburg, Sweden; 2 Department of Molecular and Clinical Medicine, Wallenberg Laboratory for Cardiovascular and Metabolic Research, University of Gothenburg, and Sahlgrenska University Hospital, Gothenburg, Sweden; 3 Cardiovascular Epidemiology, Department of Clinical Sciences in Malmö, Lund University, and Skåne University Hospital, Malmö, Sweden

## Abstract

**Background:**

Cadmium exposure may increase the risk of cardiovascular disease. The only published longitudinal study on cadmium and incident cardiovascular disease was performed in American Indians with relatively high cadmium exposure.

**Objectives:**

Our aim was to examine the association between blood cadmium at baseline and incident cardiovascular events in a population-based study of Swedish men and women with cadmium levels similar to those of most European and U.S. populations.

**Methods:**

A Swedish population-based cohort (n = 6,103, age 46–67 years) was recruited between 1991 and 1994. After we excluded those with missing data on smoking, 4,819 participants remained. Acute coronary events, other major cardiac events, stroke, and cardiovascular mortality were followed until 2010. Associations with blood cadmium (estimated from cadmium in erythrocytes) were analyzed using Cox proportional hazards regression including potential confounders and important cardiovascular risk factors.

**Results:**

Hazard ratios for all cardiovascular end points were consistently increased for participants in the 4th blood cadmium quartile (median, 0.99 μg/L). In models that also included sex, smoking, waist circumference, education, physical activity, alcohol intake, serum triglycerides, HbA1c, and C-reactive protein, the hazard ratios comparing the highest and lowest quartiles of exposure were 1.8 (95% CI: 1.2, 2.7) for acute coronary events, and 1.9 (1.3, 2.9) for stroke. Hazard ratios in never-smokers were consistent with these estimates.

**Conclusions:**

Blood cadmium in the highest quartile was associated with incident cardiovascular disease and mortality in our population-based samples of Swedish adults. The consistent results among never-smokers are important because smoking is a strong confounder. Our findings suggest that measures to reduce cadmium exposures are warranted, even in populations without unusual sources of exposure.

**Citation:**

Barregard L, Sallsten G, Fagerberg B, Borné Y, Persson M, Hedblad B, Engström G. 2016. Blood cadmium levels and incident cardiovascular events during follow-up in a population-based cohort of Swedish adults: the Malmö Diet and Cancer Study. Environ Health Perspect 124:594–600; http://dx.doi.org/10.1289/ehp.1509735

## Introduction

Exposure to the toxic metal cadmium (Cd) has adverse health effects at occupational or high-level environmental exposure. The most well-known toxic effects are kidney damage and osteoporosis/osteomalacia (Åkesson et al. 2014; Nordberg et al. 2015). In addition, cadmium is carcinogenic (IARC 2012; Nordberg et al. 2015). Cadmium contamination of agricultural soil is high in many countries, and exposure in nonsmoking populations occurs via foods such as rice, wheat, vegetables, and potatoes (WHO 2011). Tobacco smoking further increases cadmium exposure; cadmium in tobacco smoke is effectively absorbed in the lungs. The majority of circulating cadmium is bound in erythrocytes. Cadmium in blood (B-Cd) and/or urine (U-Cd) is widely used for biological monitoring (Nordberg et al. 2015; WHO 2011).

Cadmium is cytotoxic, and experimental studies indicate that cadmium exposure may cause atherosclerosis (Almenara et al. 2013; Knoflach et al. 2011; Messner et al. 2009). A systematic review and meta-analysis summarized a number of epidemiological studies on environmental exposure to cadmium and cardiovascular disease (Tellez-Plaza et al. 2013b). The pooled estimate indicated that cardiovascular disease was 36% more likely in the highest exposure category compared with the lowest. Five cohorts had examined cardiovascular mortality prospectively (Li et al. 2011; Menke et al. 2009; Nawrot et al. 2008; Tellez-Plaza et al. 2012, 2013a). The only study on incident cardiovascular disease (including nonfatal disease) was the Strong Heart Study performed in American Indians (Tellez-Plaza et al. 2013a), which showed hazard ratios (HRs) of 1.33 [95% confidence interval (CI): 1.05, 1.68) for coronary heart disease and 1.87 (95% CI: 1.22, 2.86) for stroke in the highest quartile of U-Cd (median, 1.95 μg/g creatinine) compared with the lowest (median, 0.44 μg/g). The exposure was, however, much higher than what is typical in the United States and Europe (Tellez-Plaza et al. 2012; Akerstrom et al. 2013a). The review mentioned above concluded that there is evidence supporting cadmium as an independent risk factor for cardiovascular disease, but studies on incident disease are needed (Tellez-Plaza et al. 2013b).

The aim of the present study was to examine whether cadmium exposure at baseline was associated with incident cardiovascular disease during 17 years of follow-up of a population-based study of Swedish men and women with blood cadmium levels typical for most European and U.S. populations.

## Methods

### Study Population

As previously described, all men and women 45–64 years of age, in the city of Malmö, Sweden, were invited to participate in the Malmö Diet and Cancer Study, which is investigating the relation between dietary factors and cancer (Berglund et al. 1993). Between 1991 and 1994, a random selection of half of those included in the Malmö Diet and Cancer Study, who were born between 1926 and 1945, were invited to a cardiovascular substudy; almost all agreed to participate (Hedblad et al. 2000). In total, 6,103 participants were included in the cardiovascular subcohort, and 5,540 of them provided fasting blood samples. The present study includes participants (*n* = 4,819) with data available on cadmium in blood and on smoking (see Figure S1). The study was approved by the regional ethics review board in Lund, Sweden. All participants provided written informed consent.

### Cardiovascular Risk Factors at Baseline

The participants completed questionnaires concerning lifestyle (including smoking), socioeconomic status, health, and medication (Hedblad et al. 2000). They were categorized as never-smokers, former smokers, and current smokers. Pack-years of smoking were calculated from years of smoking and the number of cigarettes smoked daily for 3,938 participants, including 95% of current smokers (1,208 of 1,276), 50% of former smokers (815 of 1,628), and all never-smokers (*n* = 1,915). Leisure time physical activity was defined as low if a participant fell in the lowest quartile of a composite measure of leisure time activities (Li et al. 2009). Low educational level was defined as < 9 years of education. Height, waist circumference, and body mass were recorded. Systolic and diastolic blood pressure were measured after supine resting for 10 min.

Overnight fasting blood samples were drawn according to standard procedures at Malmö University Hospital for determination of HDL (high-density lipoprotein) cholesterol, LDL (low-density lipoprotein) cholesterol, triglycerides, hemoglobin A1c (HbA1c), and whole blood glucose. High sensitivity C-reactive protein (CRP) was measured from frozen samples (–80°C). Participants were classified as having diabetes mellitus if they reported the diagnosis in the questionnaire, used anti-diabetic medication, or had a fasting venous whole blood glucose ≥ 6.1 mmol/L.

### End points

Outcomes were based on the *International Classification of Diseases, 9th* or *10th Revision* (ICD-9 or ICD-10) obtained from the Swedish National Hospital Discharge Register and Cause-of-Death Register, the Malmö Stroke Register, and the Swedish Coronary Angiography and Angioplasty Registry. These registries have previously been described and validated (Hammar et al. 2001; Khan et al. 2004; Ludvigsson et al. 2011; Sarno et al. 2013). The end points were as follows (ICD codes):

Acute coronary event, defined as incident acute myocardial infarction (AMI) (ICD-9: 410; ICD-10: I21) or death (only) due to ischemic heart disease (ICD-9: 412 or 414; ICD-10: I22, I23, or I25)
Major adverse coronary event, including acute coronary event, coronary artery bypass graft (CABG), and percutaneous coronary intervention (PCI). CABG and PCI were identified from national registries
Stroke (ICD-9: 430, 431, 434, 436; ICD-10: I60, I61, I63–I64)
Ischemic stroke (ICD-9: 434; ICD-10: I63)
Cardiovascular mortality (ICD-9: 390–459; ICD-10: I00–I99)
All-cause mortality (secondary end point).

The first four end points on the list above include both nonfatal and fatal events. For a specific outcome, we considered only the first event. Participants with an event before baseline were excluded in the analysis of this specific outcome. A participant could, however, contribute different events, for example first an AMI and later a stroke, or first a CABG and then an AMI. We could ascertain vital status for all participants except for 30 who emigrated during the observation period (data obtained from Statistics Sweden, based on the unique personal identification number).

### Blood Cadmium Concentrations

Both B-Cd and U-Cd reflect long-term (many years) cumulative exposure and body burden, although B-Cd is also affected by recent exposure (Nordberg et al. 2015). Blood cadmium is located in erythrocytes, and levels in plasma are marginal (Nordberg et al. 2015). Cadmium was analyzed in erythrocytes (Ery-Cd) which had been obtained by centrifugation (1,350 *g*) of whole blood collected in heparin tubes, and then kept frozen in cryotubes (Nunc®; Sigma-Aldrich) at –80°C since baseline. Cadmium in whole blood (B-Cd) was estimated by multiplying Ery-Cd by hematocrit, disregarding the minimum Cd concentration in plasma. The reason for using B-Cd as the primary Cd exposure biomarker was that most previous studies reported levels of cadmium in blood, not erythrocytes. The analysis was performed by inductively coupled plasma mass spectrometry operating in the helium collision cell mode, as described previously (Fagerberg et al. 2015). The imprecision was 9.6% (coefficient of variation), as calculated for 50 duplicate samples. The limit of detection was 0.02 μg/L; no samples had levels below this. Analysis of external quality control samples and an interlaboratory comparison showed satisfactory results (Fagerberg et al. 2015).

### Data Analyses

We studied outcomes in relation to baseline B-Cd until an event—emigration (*n* = 30) or 31 December 2010, whichever came first. Because possible effects of cadmium might not be linear, we classified B-Cd in quartiles. We assessed differences between the 4th and 1st B-Cd quartiles for baseline characteristics using a *t*-test or the Wilcoxon rank sum test. We analyzed associations between incident cardiovascular disease and baseline B-Cd using Cox proportional hazards regression with age as time scale. We estimated associations between B-Cd quartiles and cardiovascular risk factors using three models. Model 1 included B-Cd in quartiles and sex. Model 2 (main model) also included other potential confounders, as shown in Table 1: smoking, waist circumference (a marker for obesity and a strong cardiovascular risk factor), low education, low physical activity, alcohol intake, serum triglycerides, HbA1c, and CRP. Model 3 additionally included other cardiovascular risk factors not associated with B-Cd according to Table 1: postmenopausal status, hormonal replacement, treatment for hypertension, diabetes mellitus, lipid-lowering medication, diastolic blood pressure, LDL cholesterol, and HDL cholesterol.

**Table 1:** Characteristics of the study cohort at baseline by quartiles (Q) of blood cadmium.

Variable^*a*^	All	Quartiles of blood cadmium				*p*-Value Q4 vs. Q1
		1	2	3	4	
Blood cadmium quartile limits (μg/L)^*b*^		< 0.17	0.17–0.259	0.26–0.499	0.50–5.1
No. of participants	4,819	1,205	1,205	1,205	1,204
Blood cadmium [mean (median)]	0.46 (0.26)	0.13 (0.13)	0.21 (0.21)	0.35 (0.34)	1.2 (0.99)
Age (years) (mean ± SE)	57 ± 0.1	57 ± 0.2	58 ± 0.2	58 ± 0.2	57 ± 0.2	0.65
Women [*n* (%)]	2,861 (59)	597 (50)	755 (63)	796 (66)	713 (59)	< 0.001
Never-smoker [*n* (%)]	1,915 (40)	752 (62)	619 (51)	472 (39)	72 (6.0)	< 0.001
Former smoker [*n* (%)]	1,628 (34)	409 (34)	519 (43)	547 (45)	153 (13)	< 0.001
Current smoker [*n* (%)]	1,276 (26)	44 (3.7)	67 (5.6)	186 (15)	979 (81)	< 0.001
Pack-years [GM (GSD)]^*c*^	13 (3.3)	5.4 (3.9)	7.3 (3.8)	9.7 (3.2)	21 (2.3)	< 0.001
Missing data for pack-years (*n*)	681	7	272	299	103
Secondhand smoke [*n* (%)]	3,967 (73)	628 (65)	673 (68)	728 (73)	848 (84)	< 0.001
BMI (kg/m^2^) (mean ± SE)	26 ± 0.1	26 ± 0.1	26 ± 0.1	26 ± 0.1	25 ± 0.1	< 0.001
Waist (cm) [GM (GSD)]	83 (1.2)	84 (1.2)	82 (1.2)	82 (1.2)	82 (1.2)	< 0.001
Low education level [*n* (%)]	2,223 (46)	500 (42)	528 (44)	562 (47)	633 (53)	< 0.001
Low physical activity [*n* (%)]	1,115 (24)	251 (21)	268 (23)	281 (24)	315 (27)	0.001
Postmenopausal status [*n* (%)]^*d*^	2,113 (74)	443 (74)	560 (74)	590 (74)	520 (73)	0.66
Hormonal replacement [*n* (%)]^*d*^	502 (20)	112 (22)	129 (19)	142 (20)	119 (18)	0.12
Alcohol intake (g/day) (mean ± SE)	10 ± 0.2	10 ± 0.3	10 ± 0.3	10 ± 0.3	11 ± 0.4	0.05
High alcohol intake [*n* (%)]	177 (4)	45 (4)	34 (3)	34 (3)	64 (5)	0.06
Treatment for hypertension [*n* (%)]	797 (17)	180 (15)	217 (18)	223 (19)	177 (15)	0.91
Diabetes mellitus [*n* (%)]	392 (8.2)	121 (10)	92 (7.6)	83 (6.9)	96 (8.0)	0.09
Lipid-lowering treatment [*n* (%)]	117 (2.4)	28 (2.3)	23 (1.9)	38 (3.1)	28 (2.3)	1.0
Systolic blood pressure (mmHg) (mean ± SE)	141 ± 0.3	141 ± 0.5	141 ± 0.5	142 ± 0.5	140 ± 0.6	0.35
Diastolic blood pressure (mmHg) (mean ± SE)	87 ± 0.1	87 ± 0.3	87 ± 0.3	87 ± 0.3	87 ± 0.3	0.17
LDL cholesterol (mmol/L) (mean ± SE)	4.2 ± 0.01	4.2 ± 0.03	4.2 ± 0.03	4.1 ± 0.03	4.2 ± 0.03	0.09
HDL cholesterol (mmol/L) (mean ± SE)	1.4 ± 0.01	1.3 ± 0.01	1.4 ± 0.01	1.4 ± 0.01	1.3 ± 0.01	0.89
Triglycerides (mmol/L) [GM (GSD)]	1.2 (1.6)	1.2 (1.6)	1.2 (1.6)	1.2 (1.6)	1.3 (1.6)	0.03
HbA1c (mmol/mol) [GM (GSD)]	4.8 (1.1)	4.8 (1.1)	4.8 (1.1)	4.8 (1.1)	5.0 (1.1)	< 0.001
hsCRP (mg/L) [GM (GSD)]	1.4 (2.9)	1.2 (2.8)	1.2 (2.8)	1.4 (2.7)	1.8 (3.0)	< 0.001
Hematocrit (%) (mean ± SE)	41 ± 0.05	41 ± 0.1	41 ± 0.1	41 ± 0.1	42 ± 0.1	< 0.001
Abbreviations: BMI, body mass index; GM, geometric mean; GSD, geometric standard deviation; hsCRP, high-sensitivity CRP test. ^***a***^Missing data: BMI 4, waist 4, education 12, physical activity 118, menopausal status 8, hormonal replacement 317, alcohol 23, lipid-lowering treatment 136, HDL 69, HbA1c 24, hsCRP 217. The missing data were relatively evenly distributed by blood cadmium quartiles. ^***b***^1 μg/L = 9 nmol/L. ^***c***^Means refer to smoking or ex-smoking participants with available data. Data are available for 95% of current smokers and 50% of former smokers. ^***d***^Among women.						

We also investigated the effect of B-Cd as a continuous variable, using generalized additive models with the mgcv package in version 3.0.2 of the R statistical software package (R Core Team 2013). We fitted a Poisson regression model with the effect of B-Cd represented using a smooth function. The model was identical to model 2 but also included age (time scale in the Cox regression analysis). The smooth function was represented using a penalized regression spline with 3 degrees of freedom.

We examined possible effect modification for age group (> 60 years), sex, smoking (never, former, or current), education (< 9 years), waist circumference (> 85 cm), hypertension (anti-hypertensive medication), and diabetes. This was performed for acute coronary events in the main model by stratification. For smoking and sex, we also tested possible effect modification for the other outcomes. For sex, effect modification was additionally assessed by inclusion of an interaction term in the model, tested by the Wald chi-square.

We performed sensitivity analyses with the following alternative models: *a*) restriction to participants with data for all covariates, ensuring the same number of participants in all three models (*n* = 4,045); *b*) Ery-Cd instead of B-Cd; *c*) inclusion of pack-years (categorized) in the model (when data were available, *n* = 3,938); *d*) time on study as time scale (age as a covariate); *e*) sex-specific quartiles for B-Cd; *f*) inclusion of participants with an event prior to baseline; *g*) inclusion of secondhand tobacco smoke (at home or at work) as an additional predictor (when data were available); *h*) inclusion of alcohol as dichotomous variable (“high” intake defined as > 30 g/day in women and > 40 g/day in men, reported by 3.7% of participants); and *i*) inclusion of hematocrit in the models.

A two-tailed *p*-value < 0.05 was considered statistically significant.

## Results

### Baseline Characteristics

The mean estimated B-Cd level was 0.46 μg/L (median, 0.26; geometric mean, 0.31; 25th and 75th percentiles, 0.17 and 0.50 μg/L). It was 0.47 μg/L in women and 0.45 μg/L in men. The mean Cd in erythrocytes was 1.12 μg/L (1.17 in women and 1.04 in men). Hematocrit ranged from 28% to 55% (mean, 40%) in women and from 33% to 57% (mean, 43%) in men. Cardiovascular risk factors stratified by quartiles of B-Cd are shown in Table 1. As expected, smoking was strongly associated with B-Cd, and there were only 6% never-smokers in the 4th B-Cd quartile compared with 62% in the 1st quartile. The 1st quartile had the lowest fraction of women. The 4th quartile had a higher prevalence of low education and low physical activity, and higher mean levels of HbA1c and CRP. For other well-known risk factors, the differences between B-Cd quartiles were modest.

### Cardiovascular Morbidity and Mortality

From baseline to end of follow-up, there were 406 incident cases of coronary events. The analyses of coronary events were based on about 78,500 person-years. When cases of CABG or PCI were also included, there were 527 incident major adverse cardiac events. There were 346 cases of stroke, 281 of which were ischemic. In total, 882 participants died during follow-up (analyses based on approximately 81,400 person-years), and in 257 cases (29%), the cause of death was classified as cardiovascular. Numbers of first events (which were fewer than the total number of events) stratified by sex are shown in Table S1.

### Associations between Blood Cadmium and Cardiovascular Morbidity and Mortality

**Figure 1:**
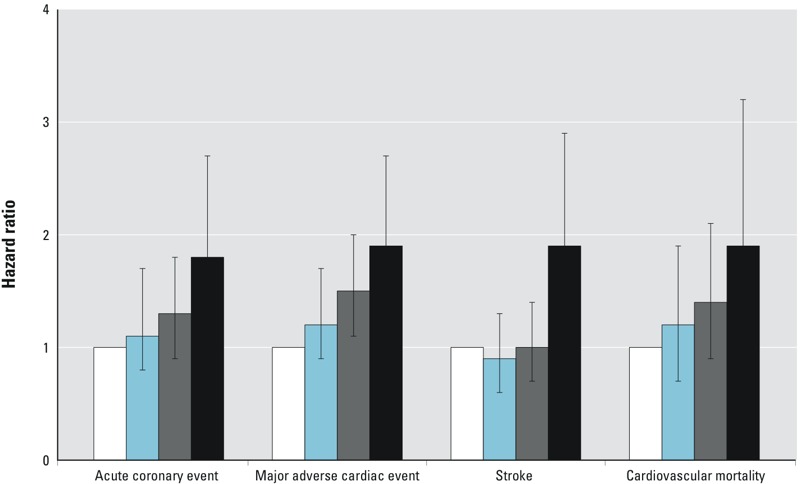
Hazard ratios and 95% confidence intervals for acute coronary event (ACE; acute myocardial infarction, or death in ischemic heart disease), major cardiac events (ACE, or coronary artery by-pass graft, or percutaneous coronary intervention), stroke, and cardiovascular mortality by quartiles of cadmium concentration in blood (white: quartile 1; blue: quartile 2; gray: quartile 3; black: quartile 4). The models were adjusted for age (time scale), sex, smoking, waist circumference, low education, low physical activity, alcohol intake, serum triglycerides, HbA1c, and C-reactive protein. For other models, see Table 2.

The HRs for all cardiovascular end points were consistently higher for participants in the 4th quartile of B-Cd (0.50–5.1 μg/L; median, 0.99 μg/L) compared with the 1st (0.02–0.17 μg/L; median, 0.13 μg/L) (Figure 1 and Table 2). The point estimate was approximately a doubled risk, regardless of the model used.

**Table 2:** Hazard ratios (95% confidence intervals) for incident first cardiovascular disease event by quartiles of cadmium concentration in blood (time scale = age).

Outcome and model	Quartiles of blood cadmium			
	1	2	3	4
Acute coronary event^*a*^
Sample size (*n *= 4,745)	1,195	1,191	1,181	1,178
No. of events (*n *= 377)	80	81	88	128
Incidence/1,000 person-years	4.0	4.0	4.1	6.9
Model 1 (377 events)	1.0	1.1 (0.8, 1.4)	1.2 (0.9, 1.6)	2.0 (1.5, 2.7)
Model 2 (328 events)	1.0	1.1 (0.8, 1.6)	1.3 (0.9, 1.8)	1.8 (1.2, 2.7)
Model 3 (306 events)	1.0	1.0 (0.7, 1.4)	1.2 (0.8, 1.6)	1.9 (1.2, 2.9)
Acute myocardial infarction
Sample size (*n *= 4,745)	1,195	1,191	1,181	1,178
No. of events (*n *= 344)	75	77	79	113
Incidence/1,000 person-years	3.7	3.8	4.0	6.1
Model 1 (344 events)	1.0	1.1 (0.8, 1.5)	1.1 (0.8, 1.6)	1.9 (1.4, 2.5)
Model 2 (305 events)	1.0	1.2 (0.8, 1.6)	1.3 (0.9, 1.8)	1.7 (1.1, 2.7)
Model 3 (283 events)	1.0	1.0 (0.7, 1.5)	1.1 (0.8, 1.6)	1.8 (1.2, 2.8)
Major adverse cardiac event^*b*^
Sample size (*n *= 4,726)	1,191	1,187	1,174	1,174
No. of events (*n *= 479)	96	193	122	158
Incidence/1,000 person-years	4.8	5.2	6.3	8.0
Model 1 (479 events)	1.0	1.2 (0.9, 1.5)	1.4 (1.1, 1.9)	2.1 (1.6, 2.7)
Model 2 (422 events)	1.0	1.2 (0.9, 1.7)	1.5 (1.1, 2.0)	1.9 (1.3, 2.7)
Model 3 (394 events)	1.0	1.1 (0.9, 1.5)	1.4 (1.0, 1.9)	1.9 (1.3, 2.8)
Any stroke
Sample size (*n *= 4,583)	1,201	1,196	1,195	1,191
No. of events (*n *= 336)	76	71	74	115
Incidence/1,000 person-years	3.8	3.5	3.7	6.2
Model 1 (336 events)	1.0	0.9 (0.6, 1.2)	0.9 (0.7, 1.3)	1.8 (1.3, 2.4)
Model 2 (294 events)	1.0	0.9 (0.6, 1.3)	1.0 (0.7, 1.4)	1.9 (1.3, 2.9)
Model 3 (271 events)	1.0	0.8 (0.6, 1.2)	0.9 (0.7, 1.4)	2.1 (1.3, 3.2)
Ischemic stroke
Sample size (*n *= 4,796)	1,202	1,201	1,196	1,197
No. of events (*n *= 278)	63	58	59	98
Incidence/1,000 person-years	3.1	2.9	2.9	5.2
Model 1 (278 events)	1.0	0.9 (0.6, 1.2)	0.9 (0.6, 1.3)	1.9 (1.3, 2.5)
Model 2 (240 events)	1.0	0.9 (0.6, 1.3)	0.9 (0.6, 1.4)	1.9 (1.2, 3.1)
Model 3 (224 events)	1.0	0.8 (0.6, 1.2)	0.9 (0.6, 1.3)	2.1 (1.3, 3.3)
All cause mortality
Sample size (*n *= 4,819)	1,205	1,205	1,205	1,204
No. of events (*n *= 882)	169	189	205	319
Mortality/1,000 person-years	8.2	9.1	10.0	16.4
Model 1 (882 events)	1.0	1.1 (0.9, 1.3)	1.2 (1.0, 1.4)	2.3 (1.9, 2.7)
Model 2 (771 events)	1.0	1.0 (0.8, 1.2)	1.1 (0.8, 1.3)	1.6 (1.2, 2.0)
Model 3 (718 events)	1.0	1.0 (0.8, 1.3)	1.0 (0.8, 1.3)	1.6 (1.3, 2.2)
Cardiovascular mortality
Sample size (*n*= 4,819)	1,205	1,205	1,205	1,204
No. of events (*n *= 257)	45	57	66	89
Mortality/1,000 person-years	2.2	2.7	3.2	4.6
Model 1 (257 events)	1.0	1.2 (0.8, 1.8)	1.4 (1.0, 2.1)	2.4 (1.7, 3.5)
Model 2 (215 events)	1.0	1.2 (0.8, 1.9)	1.4 (0.9, 2.1)	1.9 (1.1, 3.2)
Model 3 (206 events)	1.0	1.2 (0.8, 1.9)	1.3 (0.9, 2.1)	1.9 (1.1, 3.2)
Model 1 was adjusted for sex. Model 2 was also adjusted for the other potential confounders in Table 1: smoking, waist circumference, low education, low physical activity, alcohol intake, serum triglycerides, HbA1c, and CRP. Model 3 was as model 2 and also adjusted for additional cardiovascular risk factors: postmenopausal status, hormonal replacement, treatment for hypertension, diabetes mellitus, lipid-lowering medication, diastolic blood pressure, LDL cholesterol, and HDL cholesterol. Denominators are not identical for all outcomes since participants with events before baseline were excluded. ^***a***^ICD-9 codes: 410, 412, or 414 or ICD-10: I21, I22, I23, or I25. ^***b***^Acute MI, death in ischemic heart disease, coronary artery bypass graft (CABG), percutaneous coronary intervention (PCI).				

The results based on quartiles (Table 2) shows that for several of the outcomes (e.g., acute coronary event and major adverse cardiac event) HRs > 1 were found also in quartiles 2 and 3, whereas for stroke the increased risk was limited to the highest quartile. An estimate of the smoothed exposure–response relation for acute coronary event (model 2) based on B-Cd as a continuous variable is shown in Figure S2. For this outcome, the association seemed to be relatively linear up to B-Cd 1 μg/L and then flattened out.

The HRs for acute coronary events were similar when stratified by age, sex, smoking, education, waist circumference, anti-hypertensive medication, or diabetes (see Table S2). Because smoking is the most important confounder, we examined cardiovascular outcomes in the 1,915 never-smokers and found HRs in the 4th quartile similar to those in the whole study population. However, only 72 never-smokers had high B-Cd, so the number of events in this group is small (Table 3). Possible differences by sex also were studied for the other outcomes (see Table S3). Although associations were similar in women and men for acute coronary events, the HR for stroke in the highest versus lowest quartile was larger in women (HR = 2.8; 95% CI: 1.5, 5.4) than in men (HR = 1.5; 95% CI: 0.8, 2.8), and the *p*-value for interaction was 0.02.

**Table 3:** Hazard ratios for incident first cardiovascular disease by quartiles of cadmium concentration in blood (time scale = age) in never-smokers.

Outcome	Quartiles of blood cadmium			
	1	2	3	4
Acute coronary event^*a*^
Sample size (*n *= 1,782)	702	578	441	61
No. of events (*n *= 111)	47	31	26	7
HR (95% CI)	1.0	0.9 (0.6, 1.5)	1.0 (0.6, 1.6)	2.3 (1.0, 5.1)
Acute myocardial infarction
Sample size (*n *= 1,782)	702	578	441	61
No. of events (*n *= 106)	44	31	24	7
HR (95% CI)	1.0	1.0 (0.6, 1.5)	1.0 (0.6, 1.6)	2.4 (1.1, 5.4)
Major adverse cardiac event^*b*^
Sample size (*n *= 1,777)	700	576	440	61
No. of events (*n*= 136)	57	39	32	8
HR (95% CI)	1.0	1.0 (0.7, 1.5)	1.1 (0.7, 1.7)	2.2 (1.0, 4.6)
Any stroke
Sample size (*n *= 1,785)	703	579	442	61
No. of events (*n *= 111)	41	32	31	7
HR (95% CI)	1.0	1.0 (0.6, 1.6)	1.2 (0.7, 2.0)	2.2 (1.0, 4.8)
Ischemic stroke
Sample size (*n* = 1,789)	704	581	443	61
No. of events (*n *= 85)	32	25	22	6
HR (95% CI)	1.0	1.0 (0.6, 1.7)	1.1 (0.6, 2.0)	2.5 (1.0, 6.0)
All-cause mortality
Sample size (*n *= 1,793)	705	582	445	61
No. of events (*n *= 238)	93	75	59	11
HR (95% CI)	1.0	1.0 (0.7, 1.3)	1.0 (0.7, 1.3)	1.3 (0.7, 2.4)
Cardiovascular mortality
Sample size (*n*= 1,793)	705	582	445	61
No. of events (*n *= 69)	22	26	16	5
HR (95% CI)	1.0	1.4 (0.8, 2.6)	1.1 (0.6, 2.2)	2.6 (1.0, 6.9)
Model 2 is adjusted for the potential confounders in Table 1: sex, smoking, waist circumference, low education, low physical activity, alcohol intake, serum triglycerides, HbA1c, and CRP. Quartile limits are as in Table 1. ^***a***^ICD-9 codes: 410, 412, or 414 or ICD-10: I21, I22, I23, or I25. ^***b***^Acute MI, death in IHD, coronary artery bypass graft (CABG), percutaneous coronary intervention (PCI). Some individuals with CABG or PCI also developed AMI or died in IHD.				

### Sensitivity Analyses

The following sensitivity analyses, performed for model 2, all showed HRs very similar to those in Table 2:

restriction to participants with data for all covariates (*n* = 4,045; see Table S4)
Ery-Cd instead of B-Cd as measure of cadmium burden (see Table S5)
inclusion of pack-years in the model (*n* = 3,938; see Table S6)
time on study as time scale instead of age (data not shown)
sex-specific quartiles for B-Cd (data not shown)
inclusion also of participants with an event before baseline (e.g., 355 acute coronary events instead of 328; data not shown)
inclusion of second-hand tobacco smoke in the model (*n* = 3,564; data not shown)
alcohol as a dichotomous variable (data not shown)
inclusion of hematocrit in the B-Cd and Ery-Cd models (data not shown).

## Discussion

To our knowledge, this study is the first to report results on incidence of cardiovascular disease in a Caucasian population with low to moderate cadmium exposure (25th and 75th percentiles of B-Cd, 0.17 and 0.50 μg/L). These results suggest that the previous observation of elevated cardiovascular disease incidence at high cadmium exposure in Native Americans also may apply to general European and U.S. populations with lower levels of exposure. We examined cardiovascular disease incidence in 4,819 Swedish men and women over 17 years of follow-up. The incidence of most cardiovascular outcomes was doubled in the upper quartile of B-Cd (median, 0.99 μg/L) at baseline compared with the lowest quartile (median, 0.13 μg/L). HRs were 1.8 (95% CI: 1.2, 2.7) for acute coronary events and 1.9 (1.3, 2.9) for stroke in a model taking relevant confounders into account. In never-smokers, the HRs were similar, although based on few events. Because the B-Cd levels in the upper quartile of the present study (median, 0.99 μg/L) are common in many countries (Akerstrom et al. 2013a; Tellez-Plaza et al. 2012), our results suggest that cadmium may be an important cardiovascular risk factor.

### Comparison with Previous Studies

The only previous large study of incident cardiovascular disease is the Strong Heart Study in American Indians (Tellez-Plaza et al. 2013a), which reported HRs of 1.87 (95% CI: 1.34, 2.60) for cardiovascular mortality, 1.33 (95% CI: 1.05, 1.68) for incident coronary heart disease, and 1.87 for incident stroke (95% CI: 1.22, 2.86) when the highest quartile of U-Cd (median, 1.95 μg/g) was compared with the lowest (median, 0.44 μg/g). The results were thus in agreement with the present study, although at higher levels of cadmium exposure. We did not have access to U-Cd in our cohort, but levels of U-Cd in micrograms per gram creatinine are expected to be similar to (Fagerberg et al. 2012) or slightly lower than (Tellez-Plaza et al. 2012) levels of B-Cd in micrograms per liter.

Other longitudinal studies are available for cardiovascular mortality (Tellez-Plaza et al. 2013b). The largest one was performed in the NHANES 1999–2004 population with a follow-up time of 5 years; 9,000 participants showed a geometric mean B-Cd of 0.44 μg/L (Tellez-Plaza et al. 2012), only slightly higher than that in our cohort (GM 0.31 μg/L). In that study, the HR for cardiovascular mortality was 1.69 (95% CI: 1.03, 2.77), comparing the upper quintile of B-Cd (> 0.8 μg) with the lowest (< 0.22 μg/L). Results were similar for U-Cd. Thus, our results for cardiovascular mortality (HR = 1.9, 95% CI: 1.1, 3.2 in model 2, Table 2) when comparing the upper quartile (> 0.5 μg/L) with the lowest (< 0.17 μg/L) are in agreement with those of the NHANES (U.S. National Health and Examination Survey) study.

In a Japanese study of people living in a cadmium-polluted area (*n* = 3,119), the HR for cardiovascular mortality was 1.8 (95% CI: 1.0, 3.1) for men and 2.4 (95% CI: 1.1, 5.1) for women, comparing participants with U-Cd > 10 μg/g creatinine with those having U-Cd < 3.0 μg/g creatinine (Li et al. 2011). However, the analyses did not include smoking or other potential confounders.

In a Belgian study, cardiovascular mortality was followed up for 20 years in 900 people with a geometric mean B-Cd of 1.1 μg/L. The HR for cardiovascular mortality (88 cases) was 1.20 (95% CI: 0.90, 1.60) at a doubling of B-Cd (Nawrot et al. 2008).

These studies, and the present one, relied on biomarkers of cadmium exposure. There are, however, two recent reports on prospective cohort studies finding no association between cardiovascular disease and dietary cadmium intake during the past year, as estimated from food frequency questionnaires. One of the studies was performed in approximately 33,000 women (Julin et al. 2013a), and the other one in approximately 37,000 men (Julin et al. 2013a). This inconsistency may have several explanations. First, the estimated cadmium intake may suffer from errors, resulting in nondifferential misclassifications that attenuate a true relationship, as discussed by the authors. In addition, dietary intake estimates do not take into account differences in absorption of dietary cadmium attributable to, for example, varying iron stores. In fact, the Pearson correlation between estimated dietary intake in the food frequency studies and U-Cd was only 0.1. Moreover, the exposure contrast in these studies was very limited.

In Sweden, the bulk of the dietary cadmium is accounted for by cereals, potatoes, and vegetables, although higher levels are found in liver, kidney, and certain shellfish (Julin et al. 2013b).

### Potential Mechanisms

There are several potential mechanisms that could explain the association between B-Cd and cardiovascular disease found in the present study, in the NHANES study, and in the Strong Heart Study. One major hypothesis is that cadmium increases the risk of atherosclerosis (Tellez-Plaza et al. 2013b). This is supported by studies showing associations between B-Cd and the prevalence, size, and growth of atherosclerotic plaques in Swedish women (Fagerberg et al. 2012) and in the Malmö Diet and Cancer Study (Fagerberg et al. 2015), studies of cadmium and peripheral artery disease (Fagerberg et al. 2013; Tellez-Plaza et al. 2010), and a study of cadmium in plaques (Bergström et al. 2015).

There is also experimental support for a proatherogenic impact of cadmium. Cadmium exposure was associated with atherosclerosis in the aorta in ApoE knockout mice (Knoflach et al. 2011). *In vitro* studies have shown DNA damage, increased permeability of vascular endothelium, inhibition of endothelial cell proliferation, and apoptosis (Knoflach et al. 2011). Cadmium exposure may affect vascular endothelium via cellular adhesion molecules or via protein kinase signalling (Prozialeck et al. 2008). There are also experimental data indicating that cadmium exposure may have a direct toxic effect on the myocardium (Ozturk et al. 2009; Turdi et al. 2013).

### Confounding by Smoking

The point estimates of the HRs were similar in never-smokers to those in the combined group of ever- and never-smokers with smoking included in the models. This is important because confounding by smoking is always an issue in cardiovascular epidemiology. Adjustment for smoking in the statistical models may be insufficient, because self-reported smoking levels may be inaccurate. Moreover, the cadmium content in cigarette brands and the amount of smoke inhaled may differ among individuals. Such differences may be captured by biomarkers, but not by self-reported data. The meta-analysis by Tellez-Plaza et al. (2013b) found that the risk estimate for any cardiovascular disease was similar in never-smokers compared with the overall combined risk estimate, but the authors noted that more studies among never-smokers were called for.

### Strengths and Limitations

The strengths of the present prospective study are the long follow-up period (17–20 years), the fact that very few participants were lost to follow-up (30 who emigrated), and the high validity of the Swedish national death and hospital registers (Hammar et al. 2001; Khan et al. 2004; Sarno et al. 2013). For example, the sensitivity of the hospital discharge registers was 94–96% (Hammar et al. 2001). Detailed data were available on potential confounders at baseline, but we could not account for changes during follow-up. The fact that quantitative smoking data were not available for some of the former smokers is a limitation. Another limitation is that we had no access to urinary cadmium. B-Cd reflects the body burden and long-term exposure as long as dietary and smoking habits are stable, but after smoking cessation, change in dietary habits, or change in iron status, B-Cd changes faster than U-Cd (Nordberg et al. 2015). On the other hand, U-Cd may have substantial within-person variability, due to variation in renal physiology (Akerstrom et al. 2013b). The age of participants at baseline was 46–67 years. B-Cd and U-Cd normally increase with age, and in the younger participants they may have been somewhat higher in the end of the observation period than at baseline. Finally, cadmium was not determined in whole blood, but calculated from hematocrit and Cd in erythrocytes. It is not known whether Cd in whole blood or Cd in erythrocytes best reflects the body burden of Cd. High hematocrit has been reported to be a risk factor for cardiovascular disease, but we found no such impact when it was added to our models.

## Conclusion

In the present study, we have suggested that cadmium exposure is an important risk factor for cardiovascular disease. Because dietary exposure is widespread, preventive strategies in never-smokers are needed on a societal level, such as limiting industrial release of cadmium to the environment, where it ends up in cultivated land and crops. Moreover, the findings underline that continuous efforts should be made against tobacco smoking.

## Supplemental Material

(571 KB) PDF
