# Supplementary material for: Blood Cadmium Levels and Incident Cardiovascular Events during Follow-up in a Population-Based Cohort of Swedish Adults: The Malmö Diet and Cancer Study
Source: Environ Health Perspect. 2015 Oct 30;124(5):594–600. doi: 10.1289/ehp.1509735 (PMC4858401; doi:10.1289/ehp.1509735)
Supplement: (571 KB) PDF [file ehp.1509735.s001.acco.pdf]

Environ Health Perspect
DOI: 10.1289/ehp.1509735

Note to readers with disabilities: *EHP* strives to ensure that all journal content is accessible to all readers. However, some figures and Supplemental Material published in *EHP* articles may not conform to 508 standards due to the complexity of the information being presented. If you need assistance accessing journal content, please contact ehp508@niehs.nih.gov. Our staff will work with you to assess and meet your accessibility needs within 3 working days.

Supplemental Material

Blood Cadmium Levels and Incident Cardiovascular Events during Follow-up in a Population-Based Cohort of Swedish Adults: The Malmö Diet and Cancer Study

Lars Barregard, Gerd Sallsten, Björn Fagerberg, Yan Borné, Margaretha Persson, Bo Hedblad, and Gunnar Engström

Table of Contents

Table S1. Mortality and first incident cardiovascular events (*n*) during follow-up from baseline to 2010. Number of person-years (rounded) given for each analysis.

Table S2. Stratified hazard ratios (95% confidence intervals) for acute coronary event comparing the 4th quartile of B-Cd with the first quartile.

Table S3. Hazard ratios (95% CI) for first incident cardiovascular disease event by quartiles of cadmium concentration in blood **in women and men**. Quartiles of blood cadmium are based on the total data set including both men and women. The number of events (n) is given for each outcome.

Table S4. Hazard ratios (95% CI) for first incident cardiovascular disease event by quartiles of cadmium concentration in blood (time scale=age). Model 1 and 2 as in main Table 2, but **including only those with data for all covariates in Model 3, thus resulting in the same individuals in all three models (N=4045).** The number of events (n) is given for each outcome.

Table S5. Hazard ratios (95% CI) for first incident cardiovascular disease event by quartiles of **erythrocyte cadmium (µg/L) instead of blood cadmium in Model 2.** The number of events (n) is given for each outcome.

Table S6. Hazard ratios (95% CI) for first incident cardiovascular disease event by quartiles of cadmium concentration in blood. Model **additionally number of pack-years in participants with such data available (N=3938 out of 4819)**.

Figure S1. Flow chart showing the recruitment of the MDC cardiovascular cohort and the study participants of the present study.

Figure S2. Smooth function (with 95% confidence interval) describing the impact of B-Cd on the risk of acute coronary event, in a model adjusted for age, gender, smoking, waist, education, physical activity, alcohol, triglycerides, HbA1c, and C-reactive protein (=Model 2 in Table 2 + age). Graph A shows results up to B-Cd 4 μg/L Graph B shows results up to B-Cd 1 μg/L. Graph C shows results up to B-Cd 1 μg/L in women and graph D in men. Arrows indicate medians of B-Cd within quartiles.

Table S1. Mortality and first incident cardiovascular events (*n*) during follow-up from baseline to 2010. Number of person-years (rounded) given for each analysis.

Event	Total	Women	Men
All-cause mortality	882	417	465
Person-years	81400		
Cardiovascular mortality[a)]	257	113	144
Person-years	81400		
Acute coronary event	377	146	231
Person-years	78500		
Acute AMI	344	136	208
Death in IHD	33	10	23
Stroke (N)	336	170	166
Person-years	79100		
Ischemic	278	138	140
Other or unspecified	58	32	26
Major adverse cardiac event[b)]	479	180	299
Person-years	77400		
CABG	162	59	103
PCI	52	17	35

An individual could contribute to AMI as well as to stroke, and to incident disease as well as to mortality. **a)** ICD9: 390 – 459 or ICD10: I00 –I99 **b)** Acute MI, death in IHD, coronary artery bypass graft (CABG), percutaneous coronary intervention (PCI). Only the first major adverse cardiac event was considered here.

Table S2. Stratified hazard ratios (95% confidence intervals) for acute coronary event comparing the 4th quartile of B-Cd with the first quartile.[a]

Subgroup	n events/<br>N participants	HR (95% CI
**Age**		
>60 years	187/1615	1.8 (1.1 , 3.1)
≤60 years	141/2752	1.9 (0.98 , 3.6)
**Sex**		
Women	128/2628	1.7 (0.8 , 3.5)
Men	200/1739	1.9 (1.1 , 3.2)
**Smoking**		
Never	111/1782	2.3 (1.01 , 5.1)
Former	110/1463	2.1 (1.01 , 4.2)
Current	107/1122	2.3 (0.6 , 9.3)
**Education**		
<9 years	200/1974	1.8 (1.04 , 3.1)
≥9 years	128/2393	2.0 (1.03 , 3.8)
**Waist circumference (cm)**		
>85 cm	201/1812	1.9 (1.1 , 3.1)
≤85 cm	127/2555	1.8 (0.8 , 3.6)
**Antihypertensive medication**		
Yes	80/665	1.7 (0.8 , 3.8)
No	248/3702	1.9 (1.2 , 3.2)
**Diabetes**		
Yes	58/333	2.1 (0.8 , 5.7)
No	270/4034	1.8 (1.1 , 2.9)

a) Time scale is age, and the model is adjusted for sex, smoking, waist circumference, low education, low physical activity, alcohol intake, serum triglycerides, HbA1c, and CRP (=Model 2 in main Table 2).

Table S3. Hazard ratios (95% CI) for first incident cardiovascular disease event by quartiles of cadmium concentration in blood **in women and men**[a]. Quartiles of blood cadmium are based on the total data set including both men and women. The number of events (n) is given for each outcome.

	**Quartiles of blood cadmium**				**P for inter-action**
	**1**	**2**	**3**	**4**	
**Acute coronary event**					0.84
(AMI + death in IHD					
Women (n=128)	1.0	1.2 (0.7 , 2.1)	1.3 (0.7 , 2.3)	1.7 (0.8 , 3.5)	
Men (n=200)	1.0	1.1 (0.8 , 1.8)	1.4 (0.9 , 2.1)	1.9 (1.1 , 3.2)	
**Acute myocardial infarction**					0.85
Women (n=121)	1.0	1.2 (0.7 , 2.3)	1.3 (0.7 , 2.3)	1.6 (0.8 , 3.4)	
Men (n=184)	1.0	1.2 (0.7 , 1.8)	1.3 (0.9 , 2.1)	1.8 (1.1 , 3.2)	
**Major adverse cardiac event**					0.51
Women (n=161)	1.0	1.5 (0.8 , 2.6)	1.9 (1.1 , 3.3)	2.3 (1.2 , 4.5)	
Men (n=261)	1.0	1.2 (0.8 , 1.7)	1.4 (0.97 , 2.0)	1.7 (1.1 , 2.7)	
**Any stroke**					0.02
Women (n=147)	1.0	1.0 (0.6 , 1.9)	1.5 (0.9 , 2.5)	2.8 (1.5 , 5.4)	
Men (n=147)	1.0	1.0 (0.6 , 1.6)	0.7 (0.4 , 1.2)	1.5 (0.8 , 2.8)	
**Ischemic stroke**					0.007
Women (n=117)	1.0	1.1 (0.6 , 2.2)	1.7 (0.9 , 3.2)	3.1 (1.5 , 6.5)	
Men (n=123)	1.0	0.9 (0.6 , 1.5)	0.6 (0.3 , 1.04)	1.6 (0.8 , 3.0)	
**All-cause mortality**					0.68
Women (n=364)	1.0	1.0 (0.7 , 1.4)	1.0 (0.7 , 1.4)	1.8 (1.2 , 2.7)	
Men (n=407)	1.0	1.0 (0.7 , 1.4)	1.1 (0.8 , 1.5)	1.5 (1.03 , 2.1)	
**Cardiovascular mortality**					0.06
Women (n=95)	1.0	1.0 (0.5 , 1.9)	1.1 (0.5 , 2.1)	2.6 (1.2 , 5.7)	
Men (n=120)	1.0	1.5 (0.9 , 2.7)	1.8 (1.05 , 3.1)	1.6 (0.8 , 3.1)	

a) Model adjusted for smoking, waist circumference, low education, low physical activity, alcohol intake, serum triglycerides, HbA1c, CRP (=Model 2 in main Table 2).

Table S4. Hazard ratios (95% CI) for first incident cardiovascular disease event by quartiles of cadmium concentration in blood (time scale=age). Model 1 and 2 as in main Table 2, but **including only those with data for all covariates in Model 3, thus resulting in the same individuals in all three models (N=4045).** The number of events (n) is given for each outcome.

	**Quartiles of blood cadmium**			
	**1**	**2**	**3**	**4**
**Acute coronary event**				
(AMI + death in IHD)				
Model 1 (n=306)	1.0	1.0 (0.7 , 1.4)	1.2 (0.9 , 1.7)	2.1 (1.5 , 2.8)
Model 2 (n=306)	1.0	1.0 (0.7 , 1.4)	1.2 (0.8 , 1.7)	1.9 (1.2 , 2.8)
Model 3 (n=306)	1.0	1.0 (0.7 , 1.4)	1.2 (0.8 , 1.6)	1.9 (1.2 , 2.9)
**Acute myocardial infarction**				
Model 1 (n=283)	1.0	1.0 (0.7 , 1.4)	1.2 (0.8 , 1.6)	2.0 (1.4 , 2.7)
Model 2 (n=283)	1.0	1.0 (0.7 , 1.5)	1.1 (0.8 , 1.7)	2.0 (1.3 , 2.9)
Model 3 (n=283)	1.0	1.0 (0.7 , 1.5)	1.1 (0.8 , 1.6)	1.8 (1.2 , 2.8)
**Major adverse cardiac event**				
Model 1 (n=394)	1.0	1.1 (0.8 , 1.5)	1.4 (1.04 , 1.9)	2.1 (1.6 , 2.8)
Model 2 (n=394)	1.0	1.1 (0.8 , 1.5)	1.4 (1.03 , 1.9)	1.9 (1.3 , 2.8)
Model 3 (n=394)	1.0	1.1 (0.9 , 1.5)	1.4 (1.04 , 1.9)	1.9 (1.3 , 2.8)
**Any stroke**				
Model 1 (n=271)	1.0	0.9 (0.6 , 1.2)	1.0 (0.7 , 1.4)	1.9 (1.4 , 2.7)
Model 2 (n=271)	1.0	0.9 (0.6 , 1.3)	1.0 (0.7 , 1.4)	2.1 (1.3 , 3.2)
Model 3 (n=271)	1.0	0.8 (0.6 , 1.2))	0.9 (0.7 , 1.4)	2.1 (1.3 , 3.2)
**Ischemic stroke**				
Model 1 (n=224)	1.0	0.9 (0.6 , 1.3)	0.9 (0.6 , 1.3)	2.0 (1.4 , 2.8)
Model 2 (n=224)	1.0	0.9 (0.6 , 1.3)	0.9 (0.6 , 1.4)	2.1 (1.3 , 3.3)
Model 3 (n=224)	1.0	0.8 (0.6 , 1.2))	0.9 (0.6 , 1.4)	2.1 (1.3 , 3.3)
**All-cause mortality**				
Model 1 (n=718)	1.0	1.0 (0.8 , 1.3)	1.1 (0.9 , 1.4)	2.2 (1.8 , 2.7)
Model 2 (n=718)	1.0	1.0 (0.8 , 1.3)	1.0 (0.8 , 1.3)	1.7 (1.3 , 2.2)
Model 3 (n=718)	1.0	1.0 (0.8 , 1.3)	1.0 (0.8 , 1.3)	1.6 (1.3 , 2.2)
**Cardiovascular mortality**				
Model 1 (n=206)	1.0	1.3 (0.8 , 2.0)	1.5 (0.97 , 2.3)	2.5 (1.7 , 2.8)
Model 2 (n=206)	1.0	1.3 (0.8 , 2.0)	1.4 (0.9 , 2.2)	1.9 (1.1 , 3.2)
Model 3 (n=206)	1.0	1.2 (0.8 , 1.9)	1.3 (0.9 , 2.1)	1.9 (1.1 , 3.2)

Table S5. Hazard ratios (95% CI) for first incident cardiovascular disease event by quartiles of **erythrocyte cadmium (µg/L) instead of blood cadmium in Model 2.**[a)] The number of events (n) is given for each outcome.

	**Quartiles of erythrocyte cadmium**			
	**1**	**2**	**3**	**4**
**Mean**	0.31	0.52	0.85	2.8
**Range**	0.06 – 0.414	>0.41 – 0.633	>0.633 – 1.21	>1.21 – 11.3
**Acute coronary event**[b)] **(n=328, sample 4367)**	1.0	1.1 (0.8 , 1.5)	1.5 (1.04 , 2.1)	1.8 (1.2 , 2.7)
(AMI + death in IHD)				
**Acute myocardial infarction (n=305, sample 4367)**	1.0	1.1 (0.8 , 1.6)	1.5 (1.04 , 2.1)	1.7 (1.1 , 2.7)
**Major adverse cardiac event (n=422, sample 4350)**	1.0	1.2 (0.9 , 1.6)	1.6 (1.2 , 2.2)	1.7 (1.2 , 2.5)
**Any stroke (n=294, sample 4404)**	1.0	0.8 (0.6 , 1.2)	0.9 (0.6 , 1.2)	1.8 (1.2 , 2.7)
**Ischemic stroke (n=240, sample size 4416)**	1.0	0.8 (0.5 , 1.2)	0.8 (0.6 , 1.2)	1.8 (1.1 , 2.8)
**All-cause mortality (n=771, sample size 4437)**	1.0	0.9 (0.7 , 1.1)	1.0 (0.8 , 1.2)	1.5 (1.2 , 1.9)
**Cardiovascular mortality (n=215, sample size 4437)**	1.0	1.0 (0.7 , 1.6)	1.3 (0.9 , 1.9)	1.5 (0.9 , 2.5)

a) Model adjusted for sex, smoking, waist circumference, low education, low physical activity, alcohol intake, serum triglycerides, HbA1c, and CRP (=Model 2 in main Table 2).
b) ICD9: 410, 412, or 414 or ICD10: I21, I22, I23, or I25.

Table S6. Hazard ratios (95% CI) for first incident cardiovascular disease event by quartiles of cadmium concentration in blood.[a)] Model **additionally number of pack-years[b)] in participants with such data available (N=3938 out of 4819)**.

	**Quartiles of blood cadmium**			
	**1**	**2**	**3**	**4**
**Acute coronary event (n=253)**	1.0	1.0 (0.7 , 1.5)	1.2 (0.8 , 1.8)	1.8 (1.1, 2.9)
(AMI + death in IHD) [c)]				
**Acute myocardial infarction (n=238)**	1.0	1.1 (0.7 , 1.6)	1.2 (0.8 , 1.8)	1.8 (1.1 , 3.1)
**Major adverse cardiac event (n=332)**	1.0	1.1 (0.8 , 1.6)	1.4 (0.99 , 2.0)	1.8 (1.1 , 2.7)
**Any stroke (n=232)**	1.0	0.9 (0.6 , 1.3)	1.1 (0.7 , 1.6)	2.0 (1.2 , 3.4)
**Ischemic stroke (n=187)**	1.0	0.8 (0.5 , 1.3)	1.0 (0.6 , 1.5)	2.0 (1.1 , 3.6)
**All-cause mortality (n=506)**	1.0	1.0 (0.7 , 1.3)	0.9 (0.7 , 1.3)	1.5 (1.01 ,2.1)
**Cardiovascular mortality (n=140)**	1.0	1.1 (0.7 , 2.0)	1.1 (0.6 , 1.9)	1.9 (0.9 , 3.8)

a) Model adjusted for sex, smoking, waist circumference, low education, low physical activity, alcohol intake, serum triglycerides, HbA1c, CRP (=Model 2 in main Table 2), and pack-years.
b) Pack-years were categorized in five categories, one for never-smokers and four based on quartiles among ever-smokers. Category 1: 0 pack-years, Category 2: >0 but <7.5 pack-years, Category 3: ≥7.5 but <17 pack-years, Category 4: ≥17 but <30 pack-years, and Category 5: ≥30 pack-years.
c) ICD9: 410, 412, or 414 or ICD10: I21, I22, I23, or I25.

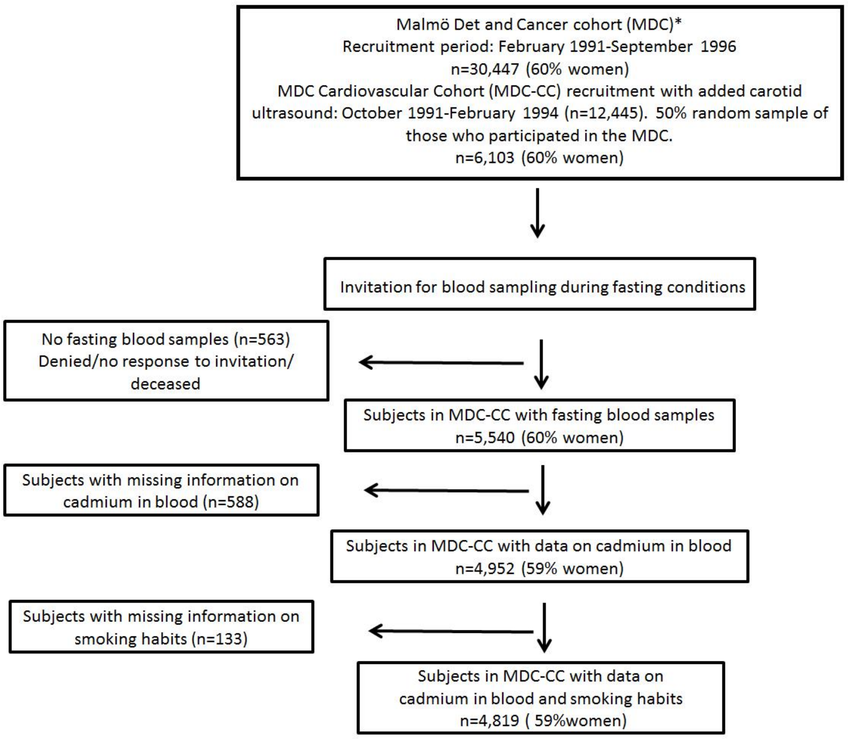

*1. Manjer J, Elmståhl S, Janzon L, Berglund G. Invitation to a population-based cohort study: differences between subjects recruited using various strategies. Scand J Public Health. 2002;30(2):103-12.
2. Manjer J, Carlsson S, Elmståhl S, Gullberg B, Janzon L, Lindström M, Mattisson I, Berglund G. The Malmö Diet and Cancer Study: representativity, cancer incidence and mortality in participants and non-participants. Eur J Cancer Prev. 2001 Dec;10(6):489-99.

Figure S1. Flow chart showing the recruitment of the MDC cardiovascular cohort and the study participants of the present study.

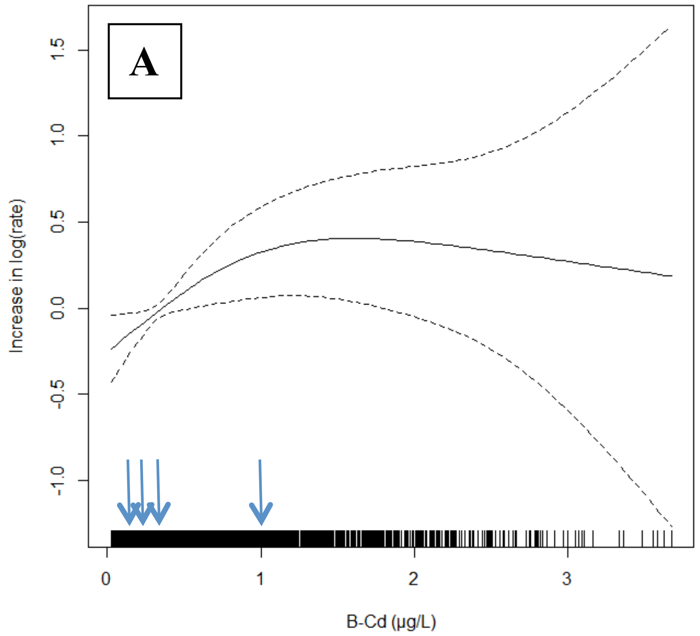
A
Increase in log(rate)
1.5
1.0
0.5
0.0
-0.5
-1.0
0
1
2
3
B-Cd (μg/L)

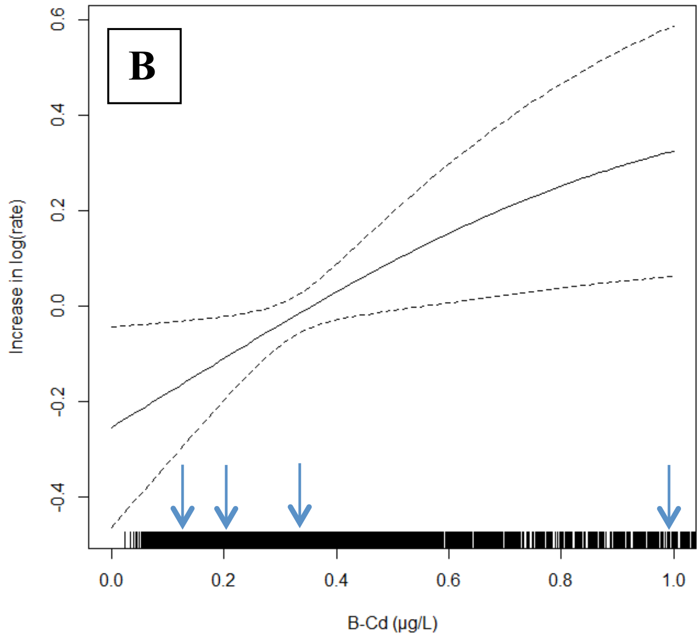
B
Increase in log(rate)
0.6
0.4
0.2
0.0
-0.2
-0.4
0.0
0.2
0.4
0.6
0.8
1.0
B-Cd (μg/L)

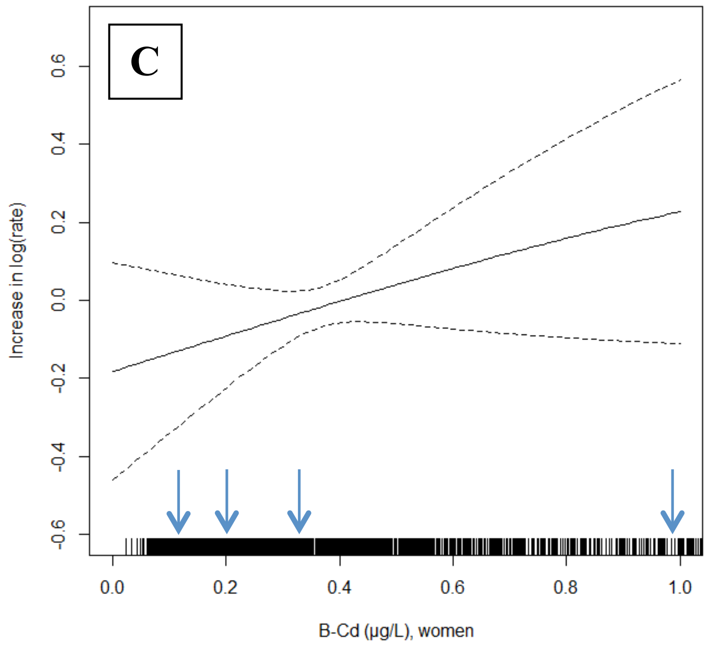

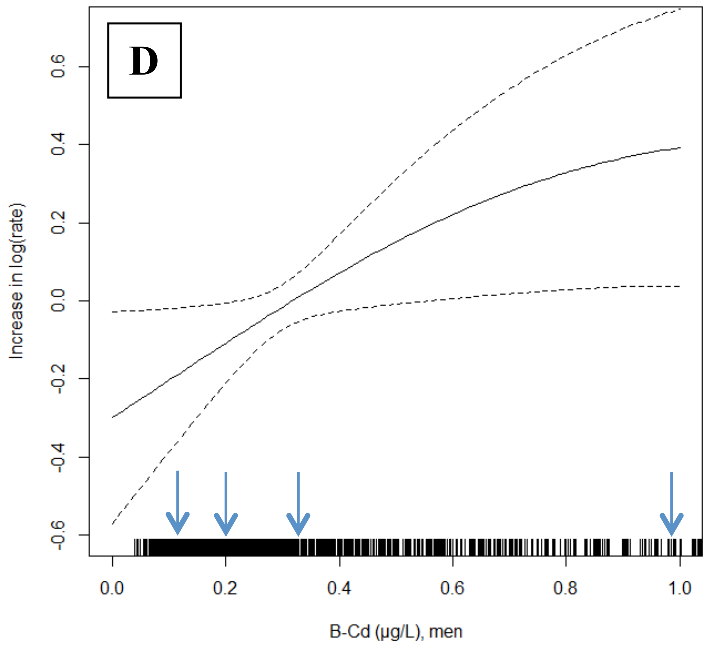

Figure S2. Smooth function (with 95% confidence interval) describing the impact of B-Cd on the risk of acute coronary event, in a model adjusted for age, gender, smoking, waist, education, physical activity, alcohol, triglycerides, HbA1c, and C-reactive protein (=Model 2 in Table 2 + age). Graph A shows results up to B-Cd 4 µg/L Graph B shows results up to B-Cd 1 µg/L. Graph C shows results up to B-Cd 1 µg/L in women and graph D in men. Arrows indicate medians of B-Cd within quartiles.
